# Left ventricular morphological and functional predictors of ${\dot{V}}_{O_{2}\mathrm{peak}}$: A 3‐year observational study

**DOI:** 10.1113/EP092871

**Published:** 2025-09-11

**Authors:** Viswanath B. Unnithan, Alexander Beaumont, Thomas Rowland, Keith George, Antonio Dello Iacono, Nicholas Sculthorpe, Rachel N. Lord, David L. Oxborough

**Affiliations:** ^1^ Sport and Physical Activity Research Institute, Division of Sport, Exercise and Health, School of Health and Life Sciences University of the West of Scotland Hamilton UK; ^2^ School of Science, Technology and Health York St. John University York UK; ^3^ Research Institute for Sport and Exercise Sciences Liverpool John Moores University Liverpool UK; ^4^ Cardiff Centre for Health, Activity and Wellbeing Research Cardiff Metropolitan University Cardiff UK

**Keywords:** adolescent, athletes, echocardiography

## Abstract

The aim of the study was to identify central determinants of ${\dot{V}}_{O_{2}\mathrm{peak}}$ using a 3‐year longitudinal evaluation of left ventricular (LV) morphological and functional (global, tissue‐Doppler and strain) outcome measures obtained at rest and during both submaximal and maximal exercise in a group of highly trained male youth soccer players (SP) and recreationally active male participants (CON). Once a year for 3 years, measurements were obtained in both the SP and CON groups (12.0 ± 0.3 and 11.7 ± 0.2 years of age, respectively, at the onset of the study). Cardiac ultrasound measures were used to identify LV morphological indices at rest and functional parameters during submaximal and maximal exercise. Training status (*P* < 0.0001) emerged as the only significant independent predictor of ${\dot{V}}_{O_{2}\mathrm{peak}}$, when considering LV morphological variables. At maximal exercise, early diastolic filling (*E*) was a significant (*P* = 0.001) predictor of ${\dot{V}}_{O_{2}\mathrm{peak}}$, irrespective of the influence of training status. Training status emerged as the significant predictor of ${\dot{V}}_{O_{2}\mathrm{peak}}$ across all models that were developed in this study. Minimal LV structural and functional adaptations at both rest and exercise influence ${\dot{V}}_{O_{2}\mathrm{peak}}$, beyond the impact of training status alone. The broader implication of these findings is that the influence of LV cardiac adaptations on ${\dot{V}}_{O_{2}\mathrm{peak}}$ over time is mediated by the stimulus of training; this association occurs independently from the impact of growth and maturation on ${\dot{V}}_{O_{2}\mathrm{peak}}$.

## Abstract (highlights)

- **What is the central question of this study?**
  What is the influence of training and growth and maturation on the predictive power of cardiac determinants on aerobic capacity measured at rest and during exercise in a group of highly trained adolescent soccer players?
- **What is the main finding and its importance?**
  The training stimulus associated with high‐volume soccer training emerged as the primary determinant of maximal aerobic capacity (${\dot{V}}_{O_{2}\mathrm{peak}}$) and cardiac adaptation beyond any influence of growth and maturation. This highlights the positive benefits that high‐volume training can confer on the young athlete.

## 1 INTRODUCTION

Within the sport of soccer, it has been identified that maximal oxygen uptake (${\dot{V}}_{O_{2}\max}$) is positively related to in situ markers of training load such as total distance covered, high speed running and very high‐speed running in pre‐peak height velocity (PHV) and circa‐PHV players (Doncaster, 2018). Cardiac remodelling and increased cardio‐respiratory fitness adaptations in response to training are factors which will enhance oxygen delivery, peripheral extraction and ultimately ${\dot{V}}_{O_{2}\max}$ (Armstrong et al., 2015; Lundby et al., 2017).

There is a paucity of studies exploring the longitudinal evolution of key cardiac determinants of the oxygen delivery transport chain in trained and untrained adolescents, where the complex interaction between growth, maturation and training could modify the influence of these determinants. The limited studies that do exist in this area have demonstrated an array of bi‐variate correlations between volumetric cardiac indices, such as left ventricular end diastolic volume (LVEDV) and ${\dot{V}}_{O_{2}\max}$ in adolescents ranging from *r* = 0.67–0.85 (Forså et al., 2023) to *r* = 0.3 (Ujvári et al., 2024). While these findings provide insight, they are limited by the lack of consideration of growth and maturation on these relationships.

Adopting a stepwise, multiple linear mixed effects analytical approach allows for a more comprehensive understanding of the variables that are related to variance in ${\dot{V}}_{O_{2}\max}$. Seminal work by Perkins et al. (2022), in a cross‐sectional analysis of endurance trained and untrained males, demonstrated that prior to PHV, only LVEDV explained a significant proportion of the variance (22%) in ${\dot{V}}_{O_{2}\max}$. Post‐PHV, LVEDV and inter‐ventricular septal thickness (VSd) emerged as significant predictors of ${\dot{V}}_{O_{2}\max}$. Based on these findings and bivariate correlations between haemoglobin mass (*r* = 0.54; *P *< 0.01) and blood volume measures (*r* = 0.49; *P *< 0.01) with ${\dot{V}}_{O_{2}\max}$, the authors stated that cardiac and haematological measures in concert provided significant independent predictors of ${\dot{V}}_{O_{2}\max}$ post‐PHV compared to pre‐PHV. This suggests that there may be a maturation‐dependent shift toward more central determinants of the oxygen delivery chain in post‐PHV males (Perkins et al., 2022).

The research in this area, while providing interesting insights into the key cardiac determinants of ${\dot{V}}_{O_{2}\max}$, is somewhat limited by the cross‐sectional study designs (Forså et al., 2023; Perkins et al., 2022; Ujvári et al., 2024). Previous work from our research team has generated novel longitudinal (3 years) data on the effect of high‐volume soccer training on left ventricular (LV) structural and functional adaptations in a group of highly trained soccer players (SP) and age matched controls, as they transitioned from the pre‐PHV to the circa‐PHV phase of their biological development (Unnithan et al., 2022, 2018, 2024). A further unique element of this work was the capture of cardiac data during exercise as well as at rest. Systolic and diastolic function and cardiac strain were acquired at submaximal and maximal exercise to provide a deeper interrogation of LV function during exercise (Unnithan et al., 2022, 2018). This collective body of work demonstrated that after controlling for the effect of growth (pre‐PHV to circa‐PHV), there existed a training volume threshold over the 3 years that was the major determinant of the structural cardiac adaptations at rest (Unnithan et al., 2024) and functional adaptations during exercise (Unnithan et al., 2022) noted in these highly trained SPs.

The evidence from the previous body of work identified morphological and functional LV adaptations at rest and during exercise over a 3‐year period in the SPs in response to a training stimulus; but it did not couple these adaptations to any potential benefit for functional capacity (${\dot{V}}_{O_{2}\mathrm{peak}}$) and ultimately soccer performance in the SPs. A deeper interrogation of this research question over a key training and growth period for these SPs will have the potential to provide insight for scientists and practitioners working within soccer on the predictive power of cardiac structure and function metrics on physical performance capacity in pre‐PHV and circa‐PHV players.

Consequently, the aim of the present investigation was to identify central determinants of ${\dot{V}}_{O_{2}\mathrm{peak}}$ using a longitudinal (3 years) evaluation of LV structural and functional (global, tissue‐Doppler and strain) outcome measures at rest and during both submaximal and peak exercise in a group of highly trained SPs and recreationally active male participants (CON) as they transitioned from pre‐PHV to circa‐PHV. It was hypothesized that morphological and functional adaptations of the LV in response to prolonged training (3 years) in the SPs would explain a large proportion of the variance in ${\dot{V}}_{O_{2}\mathrm{peak}}$ in a group of SPs and CON.

## 2 METHODS

### 2.1 Ethical approval

Written assent was provided by both SP and CON participants and written informed consent was provided by all parents/legal guardians. All procedures performed in the study were in accordance with the ethical standards of the *Declaration of Helsinki* and the study was approved by Staffordshire University Ethics Committee.

### 2.2 Participants

Twenty‐two highly trained male youth SPs from two Category 1 (highest level) English Premier League Academy Under‐12 (U‐12) teams (age: 12.0 ± 0.3 years at the onset of the study) were evaluated for morphological and functional characteristics of the LV. In the U‐12 age group, players at both clubs trained for 7.3 h per week including matches. The training volume increased to 10.5 h per week at both Under‐13 (U‐13) and Under‐14 (U‐14) level. This was supplemented by 2.6 h per week of physical education and sports activities away from the football clubs and this remained constant over the 3 years. Data were obtained from the training database of the two clubs. Assessments were made at rest and during submaximal and maximal exercise intensities on a cycle ergometer once a year, for three consecutive soccer seasons as the players progressed from the U‐12 to U‐14 teams within their respective clubs. Furthermore, oxygen uptake was measured at both submaximal and peak exercise intensities on the cycle ergometer at the same visits. Simultaneously, a group of 15 recreationally active (age: 11.7 ± 0.2 years at the onset of the study), but not systematically trained control participants (CON) were evaluated using the identical protocols at the same time point during the year over the same 3‐year duration. The CON group underwent 3.5 h per week of physical education classes, and this remained constant across the years. Inclusion criteria for the control group were that they were recreationally active, but not involved in any systematic training. Physical activity levels were obtained from a validated questionnaire. Any participant, in either group, having any type of lower‐limb orthopaedic injury was excluded from the study (Unnithan et al., 2022). Anthropometric measures including stature, sitting height and body mass were obtained. In order to obtain a somatic marker of growth and maturation, maturity status was subsequently quantified using the Sherar et al. (2005) maturity offset method.

### 2.3 Resting and exercise measurements

Resting arterial blood pressure was recorded in the left arm by an automated blood pressure cuff (Boso, Medicus, Jungingen, Germany) and heart rate was assessed by a 12‐lead electrocardiogram (ECG) (CardioExpress SL6, Spacelabs Healthcare, Snoqualmie, WA, USA). Resting LV morphological and functional echocardiographic measurements were taken in a supine position. Participants then completed a cycle ergometer test to volitional exhaustion, with LV functional echocardiographic and open circuit, breath‐by‐breath metabolic measurements (Cortex MetaMax 3B, Cortex Biophysik GmbH, Leipzig, Germany) obtained throughout. The participant cadence on the cycle ergometer was 60 rpm with an initial workload of 20 W, with two subsequent submaximal 20 W increments to achieve workloads of 40 and 60 W. Each stage was 3 min in duration. Functional echocardiographic measurements were taken 90 s into each stage for the first three stages (20, 40 and 60 W). After the third stage, the incremental increases in workload were adjusted on an individual basis until volitional exhaustion. Final functional echocardiographic measurements were taken immediately prior to peak exercise. Peak exercise was determined by both volitional signs of exhaustion (facial flushing and inability to maintain power output) and a heart rate exceeding 180 beats/min (Unnithan et al., 2018).

### 2.4 Echocardiographic measurements: Indices of LV structure at rest

All morphological echocardiographic data‐capture procedures over the 3‐year period were performed by the same experienced cardiac echo‐sonographer (D.O.) using a commercially available ultrasound system (VividQ Ultrasound System, GE Healthcare, Horton, Norway). Two‐dimensional images from the apical and parasternal views (long and short axis) were obtained with the participants in the left lateral decubitus position. Images were subsequently stored for offline data analyses (Echopac, Version 6.0, GE Healthcare, Horton, Norway). Detailed information on the methodological approaches used to capture and scale the LV morphological data in both the SP and CON groups across the 3‐year period can be found in Unnithan et al. (2024). The variables were LV end diastolic dimension (LVED), LV end systolic dimension (LVES), diastolic wall thicknesses [interventricular septum (VSd), posterior wall (PWd)], LV mass (LV mass index), relative wall thickness (RWT), LV end‐diastolic volume (LVEDV) and LV end‐systolic volume (LVESV).

### 2.5 Echocardiographic measurements: Indices of LV function at rest

Detailed information on the methodological approaches used to capture the LV functional data [peak early diastolic filling velocity (*E*); peak longitudinal mitral and lateral annular velocities during systole (*S*′) and early diastole (*E*′); and *E*/*E*′; ejection fraction (LVEF)] in both the SP and CON groups across the 3‐year period can be found in Unnithan et al. (2024).

A sub‐group of five SPs in the third year of the study were asked to return to the football club 7 days after the third‐year cardiac evaluation to establish the test–retest reliability of LV functional indices. Coefficients of variation (CV) were: *S*′, 9.1%; *E*, 5.8%; *E*′, 16.2%; and *E*/*E*′, 11.9%.

### 2.6 Echocardiographic measurements: Indices of LV function during incremental exercise

Following supine, resting LV morphological and functional measurements, participants sat in an upright position on an electronically braked cycle ergometer (Lode, Corival, Groningen, Netherlands). Imaging of the LV was performed by the same researcher (D.O.) at rest and at 90 s into each of the first three stages, and the last 30 s of the final stage. Detailed information on the methodological approaches used to capture the pulsed‐wave and tissue Doppler LV functional data during submaximal and maximal exercise can be found in Unnithan et al. (2022, 2018).

### 2.7 Strain data acquisition during exercise

Detailed information on the methodological approaches used to capture the cardiac strain data during submaximal exercise [peak longitudinal strain (ε), systolic strain rate (SSR) and early diastolic strain rate (DSR)] during submaximal exercise can be found in Unnithan et al. (2022). Good reliability of the in‐exercise peak longitudinal ε data has also been previously established by this research team (Unnithan et al., 2015).

### 2.8 Gas exchange measurements during exercise

Detailed information on the methodological approaches used to obtain the gas exchange measurements can be seen in Unnithan et al. (2018).

### 2.9 Statistical analysis

To examine the effects of the structural and functional cardiac predictors on ${\dot{V}}_{O_{2}\mathrm{peak}}$ outputs, we fitted a series of linear mixed effects models as follows:

$${\dot{V}}_{O_{2}\mathrm{peak}}in=b_{0in}+b_{1-n}\mathrm{covariates}+\varepsilon_{i}$$

${\dot{V}}_{O_{2}\mathrm{peak}}$ represented the repeated‐measures outcome for the participant*_in_* and the number of the covariates 1 − *n* varied depending on the model and included: TrUtr (categorical variable with 2 levels, Trained, Untrained), maturity offset, body mass, PWd, VSd, RWT, LV mass index, RHR, LVEF, SVIndex, *S*′_adj_, *E*, *E*′, *E*/*E*′_delta_, *E*′_adjdelta_, *S*′_adjdelta_, *E*/*E*′_max_, *E*′_adjmax_, *S*′_adjmax_, peak ε, SSR and DSR (all continuous variables). Categorical and continuous variables were modelled as fixed effects. For the categorical predictor TrUtr (training status), the level Untrained was fitted in the model as reference. Moreover, random effects were assumed for Participants, with random intercepts introduced in the model to account for the dependence between repeated measures. For the evaluation of the linear mixed effects models, restricted maximum likelihood estimation was used since both fixed and random effects were included in the models. The contribution of both fixed and random effects to the explanatory power was examined using a likelihood ratio test, deviance statistic, Akaike information criterion score, and absence of multicollinearity, before selecting the final model to obtain the best fit while maintaining model parsimony. The selection of covariates included in the final models was based on a combination of conceptual reasoning and a backward elimination approach. Initially, the set of covariates was determined through discussion among the authors, drawing on domain expertise and consideration of the physiological mechanisms underpinning the associations between the covariates and the outcomes of interest. Subsequently, covariates were removed sequentially according to backward elimination, guided by assessments of multicollinearity. Specifically, a variance inflation factor (VIF) >3 was used as the cut‐off to identify and exclude covariates with high collinearity (Zuur et al., 2010). The statistical significance of fixed effects was set at *P *< 0.05 and examined by *t*‐tests based on the Satterthwaite approximation. All statistical analyses were conducted in R language and environment for statistical computing using the *emmeans*, *ggeffects, lme4*, and *sjPlot* packages while model assumptions were checked using the *performance* package (4.0.5; R Core Team, Vienna, Austria).

## 3 RESULTS

Physical characteristics of the SP and CON groups across all three years are highlighted in Table 1. All linear mixed effects models included training status, body mass and maturity offset unless collinearity existed with selected structural and global functional independent variables. Furthermore, collinearity within selected tissue Doppler imaging (TDI) variables used in the models led to the non‐inclusion of certain markers of systolic and diastolic function in the respective models.

**TABLE 1:** Physical characteristics of the SP and CON across the 3 years of the study.

	Soccer players			Control		
	Year 1	Year 2	Year 3	Year 1	Year 2	Year 3
Maturity offset (years)	−2.1 ± 0.58	−1.1 ± 0.56	−0.5 ± 0.69	−2.4 ± 0.48	−2.5 ± 0.48	−0.5 ± 0.72
Age (years)	12.0 ± 0.3	13.0 ± 0.3	13.9 ± 0.3	11.7 ± 0.2	12.6 ± 0.1	13.6 ± 0.2
Stature (cm)	151.3 ± 6.3	156.1 ± 7.8	164.3 ± 8.8	146.8 ± 6.4	152.4 ± 5.9	161.9 ± 6.9
Body mass (kg)	40.2 ± 5.8	45.4 ± 6.6	50.7 ± 7.6	43.3 ± 12.1	48.2 ± 12.4	57.8 ± 14.7
${\dot{V}}_{O_{2}\mathrm{peak}}$ (mL/kg/min)	47.7 ± 4.7	46.5 ± 4.7	47.9 ± 4.3	40.1 ± 7.5	37.2 ± 6.6	37.3 ± 9.4

All values are means ± SD.

### 3.1 Structural and functional independent variables at rest

Four linear mixed effects models were created for structural and functional independent variables derived at rest across the three years. The following independent variables were included in individual mixed effect models: PWd (*n* = 40), VSd (*n* = 40) and RWT (*n* = 40). LVMI, RHR, LVEF and SVIndex (*n* = 40) were collectively grouped in one model. Across all four models, training status (PWd: *P* < 0.0001; VSd: *P* < 0.0001; RWT: *P* < 0.0001; and LVMI, RHR, LVEF and SVIndex: *P* < 0.0001) emerged as the only significant independent predictor of ${\dot{V}}_{O_{2}\mathrm{peak}}$. Furthermore, maturity offset was not a significant predictor of ${\dot{V}}_{O_{2}\mathrm{peak}}$ in any of the four models (PWd model: *P *= 0.534; VSd model: *P *= 0.555; RWT model: *P *= 0.423; and LVMI, RHR, LVEF and SVIndex model: *P *= 0.140). The cumulative effect of all the independent variables included in the respective models accounted for approximately 83% of the variability in ${\dot{V}}_{O_{2}\mathrm{peak}}$.

### 3.2 Submaximal exercise independent variables

Three linear mixed effects models (*n* = 38) were created for TDI and pulsed‐wave Doppler independent variables measured at three submaximal exercise intensities (20, 40 and 60 W) for the SP and CON groups across the three years. The following independent variables were included in each model: *S*′_adj_, *E* and *E*/*E*′. Across all three models at 20, 40 and 60 W, training status (20 W model: *P *= 0.0003; 40 W model: *P *= 0.0005; and 60 W model: *P *= 0.0006) and body mass (20 W model: *P *= 0.0005; 40 W model: *P *= 0.0010; and 60 W model: *P *= 0.0008) emerged as significant independent predictors of ${\dot{V}}_{O_{2}\mathrm{peak}}$. Maturity offset was a significant predictor at 20 W (*P *= 0.046) and 40 W only *(P *= 0.046). *S*′_adj_, *E* and *E*/*E*′ were not significant predictors of ${\dot{V}}_{O_{2}\mathrm{peak}}$ in any of the three models (20 W model: *S*′_adj_: *P *= 0.208; *E*: *P *= 0.526; and *E*/*E*′: *P *= 0.669; 40 W model: *S*′_adj_: *P *= 0.358; *E*: *P *= 0.247; and *E*/*E*′: *P *= 0.326; and 60 W model: *S*′_adj_: *P *= 0.582; *E*: *P *= 0.753; and *E*/*E*′: *P *= 0.933). The cumulative effect of all the independent variables included in the respective models accounted for approximately 84% of the variability in ${\dot{V}}_{O_{2}\mathrm{peak}}$ (Table 2).

**TABLE 2:** Linear mixed effects model for TDI adjusted and pulsed‐wave Doppler independent variables at 20 W.

	${\dot{V}}_{O_{2}\mathrm{peak}}$ (mL/kg/min)		
Predictor	Estimate	CI	*P*
(Intercept)	66.05 ± 7.73	50.65 to 81.45	**<0.0001**
TrUtr [trained]	7.19 ± 1.91	−10.98 to −3.39	**0.0003**
Body mass (kg)	−0.39 ± 0.11	−0.61 to −0.18	**0.0005**
Maturity offset (years)	1.73 ± 0.85	0.03 to 3.43	**0.046**
*S*′_adj_ (cm/s/mm)	20.19 ± 15.9	−51.86 to 11.48	0.208
*E* (cm/s)	0.03 ± 0.04	−0.06 to 0.12	0.526
*E*/*E*′	0.17 ± 0.39	−0.61 to 0.95	0.669
Random effect			
σ^2^	8.73		
τ_00code_	22.54		
ICC	0.72		
*n*	39		
Observations	82; marginal *R* ^2^/conditional *R* ^2^: 0.480/0.855		

All estimate values are means ± SD. *E*, peak early diastolic filling velocity; *E*′, early peak longitudinal mitral annular velocity in diastole; *E*/*E*′, calculated as an estimate of LV filling pressure and thus preload; *S*′_adj_, peak longitudinal mitral annular velocity in systole adjusted for LV length.

### 3.3 TDI delta and pulsed‐wave Doppler independent variables (Peak − Rest)

One linear mixed effects model (Table 3; *n* = 40) was created for TDI and pulsed‐wave Doppler independent variables used to derive delta (peak − rest). The following independent variables were included in this model (Table 3): *S*′_adjdelta_, *E*_delta_, *E*′_adjdelta_ and *E*/*E*′_delta_. Training status (*P* < 0.0001) and body mass (*P *= 0.0008) were independent predictors of ${\dot{V}}_{O_{2}\mathrm{peak}}$. Irrespective of training status, *E*_delta_ (*P* = 0.038) and *E*/*E*′_delta_ (*P *= 0.004) were also significant predictors of ${\dot{V}}_{O_{2}\mathrm{peak}}$. Furthermore, maturity offset (*P *= 0.114) and *S*′_adjdelta_ (*P *= 0.598) were not significant predictors of ${\dot{V}}_{O_{2}\mathrm{peak}}$ (Table 3). The cumulative effect of all the independent variables included in the model accounted for approximately 84% of the variability in ${\dot{V}}_{O_{2}\mathrm{peak}}$ (Table 3).

**TABLE 3:** Linear mixed effects model for TDI and pulsed‐wave delta (Peak − Rest).

	${\dot{V}}_{O_{2}\mathrm{peak}}$ (mL/kg/min)		
Predictor	Estimate	CI	*P*
(Intercept)	64.54 ± 5.91	52.79 to 76.29	**<0.0001**
TrUtr [trained]	8.13 ± 1.77	−11.65 to −4.61	**<0.0001**
Body mass (kg)	−0.35 ± 0.10	−0.55 to −0.15	**0.0008**
Maturity Offset (years)	1.19 ± 0.74	−0.29 to 2.66	0.114
*E*_delta_ (cm/s)	0.05 ± 0.02	0.00 to 0.10	**0.038**
*E*/*E*′_delta_	−0.73 ± 0.25	−1.22 to −0.23	**0.004**
*E*′_adjdelta_ (cm/s/mm)	−20.55 ± 10.7	−41.79 to 0.69	0.058
*S*′_adjdelta_ (cm/s/mm)	−5.57 ± 10.5	−26.47 to 15.33	0.598
Random effect			
σ^2^	9.11		
τ_00code_	20.59		
ICC	0.69		
*n*	40		
Observations	93; marginal *R* ^2^/conditional *R* ^2^: 0.476/0.839		

All estimate values are means ± SD. *E*_delta_, early diastolic filling velocity: peak − resting values; *E*’_adjdelta_, longitudinal mitral annular velocity in diastole adjusted for LV length (*E*′_adjdelta_): peak − resting values; *E*/*E*’_delta_, LV filling pressure: peak − resting values; *S*′_adjdelta_, longitudinal mitral annular velocity in systole adjusted for LV length: peak − resting values.

### 3.4 Pulsed‐wave Doppler independent variables at peak exercise

One linear mixed effects model (Table 4; *n* = 40) was created for TDI and pulsed‐wave Doppler independent variables measured at peak exercise. The following independent variables were included in the linear mixed effects model (Table 4): *S*′_adjmax_, *E*_max_, *E*′_adjmax_ and *E*/*E*′_max_. Training status (*P* = 0.0002) and body mass (*P *< 0.0001) were independent predictors of ${\dot{V}}_{O_{2}\mathrm{peak}}$. Irrespective of training status, *E*_max_ (*P *= 0.001) was also a significant predictor of ${\dot{V}}_{O_{2}\mathrm{peak}}$. Furthermore, maturity offset (*P *= 106), *S*′_adjmax_ (*P *= 963), *E*′_adjmax_ (*P *= 0.059) and *E*/*E*′_max_ (*P *= 0.067) were not significant predictors of ${\dot{V}}_{O_{2}\mathrm{peak}}$ (Table 4). The cumulative effect of all the independent variables included in the respective models accounted for approximately 84% of the variability in ${\dot{V}}_{O_{2}\mathrm{peak}}$ (Table 4).

**TABLE 4:** Linear mixed effects model for maximal exercise TDI and pulsed‐wave Doppler data.

	${\dot{V}}_{O_{2}\mathrm{peak}}$ (mL/kg/min)		
Predictors	Estimates	CI	*P*
(Intercept)	66.38 ± 11.23	44.05 to 88.71	**<0.0001**
TrUtr [trained]	6.26 ± 1.60	−9.45 to −3.06	**0.0002**
Body mass (kg)	−0.45 ± 0.10	−0.65 to −0.25	**<0.0001**
Maturity offset (years)	1.25 ± 0.77	−0.27 to 2.78	0.106
*E*_max_ (cm/s)	0.19 ± 0.06	0.08 to 0.31	**0.001**
*E*/*E*′_max_	−2.24 ± 1.21	−4.64 to 0.16	0.067
*E*′_adjmax_ (cm/s/mm)	−46.86 ± 24.5	−95.55 to 1.82	0.059
*S*′_adjmax_ (cm/s/mm)	−0.50 ± 10.6	−21.50 to 20.50	0.963
Random effects			
σ^2^	10.82		
τ_00code_	13.49		
ICC	0.55		
*n*	40		
Observations	93; marginal *R* ^2^/conditional *R* ^2^: 0.566/0.807		

All Estimate values are means ± SD. *E*_max_, peak early diastolic filling velocity at maximal exercise intensity; *E*′_adjmax_, peak longitudinal mitral annular velocity in diastole adjusted for LV length; *E*/*E*′_max_, LV filling pressure at maximal exercise intensity; *S*′_adjmax_, peak longitudinal mitral annular velocity in systole adjusted for LV length at maximal exercise intensity.

### 3.5 Submaximal cardiac strain independent variables

Three linear mixed effects models were created for strain independent variables measured at three submaximal exercise intensities (20 W (*n* = 37), 40 W (*n* = 35) and 60 W (*n* = 37)) for the SP and CON groups across the three years. The following independent variables were included in each linear mixed effects model: peak ε, SSR and DSR. At 20 W (*P *= 0.0004), 40 W (*P *= 0.0001) and 60 W (*P *= 0.0003), training status was identified as a significant independent predictor of ${\dot{V}}_{O_{2}\mathrm{peak}}$. A similar pattern was noted for body mass at 20 W (*P *= 0.009) and 40 W (*P *= 0.039). Regardless of the influence of training status, SSR at 20 W was a significant (*P *= 0.03) predictor of ${\dot{V}}_{O_{2}\mathrm{peak}}$ (Table 5). Furthermore, maturity offset and peak ε at all three submaximal workloads, SSR (40 and 60 W) and DSR (20, 40 and 60 W) were not significant predictors of ${\dot{V}}_{O_{2}\mathrm{peak}}$ in any of the three models (20 W model: maturity offset: *P *= 0.328; peak ε: *P *= 0.224; and DSR: *P *= 0.315; 40 W model: maturity offset: *P *= 0.465; peak ε: *P *= 0.631; SSR: *P *= 0.883; and DSR: *P *= 0.233; 60 W model: maturity offset: *P *= 0.899; peak ε: *P *= 0.123; SSR: *P *= 0.059; and DSR: *P *= 0.148) as was body mass at 60 W (*P *= 0.094). The cumulative effect of all the independent variables included in the respective models accounted for approximately 78% of the variability in ${\dot{V}}_{O_{2}\mathrm{peak}}$

**TABLE 5:** Linear mixed effects model for cardiac strain data at 20 W.

	${\dot{V}}_{O_{2}\mathrm{peak}}$ (mL/kg/min)		
Predictors	Estimates	CI	*P*
(Intercept)	68.17 ± 9.32	49.54 to 86.81	**<0.0001**
TrUtr [trained]	7.02 ± 1.89	−10.78 to −3.25	**0.0004**
Body mass (kg)	−0.35 ± 0.13	−0.60 to −0.09	**0.009**
Maturity offset (years)	1.09 ± 1.10	−1.12 to 3.30	0.328
Peak ε (%)	−0.37 ± 0.30	−0.98 to 0.23	0.224
SSR (1/s)	6.85 ± 3.09	0.68 to 13.02	**0.030**
DSR (1/s)	−1.41 ± 1.39	−4.19 to 1.37	0.315
Random effects			
σ^2^	11.96		
τ_00code_	16.32		
ICC	0.58		
*n*	37		
Observations	73; marginal *R* ^2^/conditional *R* ^2^: 0.483/0.781		

All estimate values are means ± SD. DSR, diastolic strain rate; ε, peak longitudinal strain; SSR, systolic strain rate.

## 4 DISCUSSION

The aim of this investigation was to use longitudinal structural and functional cardiac outcome measures at rest and during exercise collected over a 3‐year period to elucidate predictors of ${\dot{V}}_{O_{2}\mathrm{peak}}$ in a group of highly trained and recreationally active pre‐PHV and circa‐PHV males. The longitudinal design, *in‐exercise* collection of pulsed‐wave, TDI and cardiac strain data, and a control group of age‐matched, recreationally active participants positions this work as both rigorous and unique within the field.

Training status was a significant predictor of ${\dot{V}}_{O_{2}\mathrm{peak}}$ across the three years in most of the structural and functional linear mixed effects models. It is likely, however, that structural and functional characteristics of the LV contributed to the development of ${\dot{V}}_{O_{2}\mathrm{peak}}$ across the three years, but their impact was subsumed within the domain of the training status independent variable; consequently, our hypothesis was partially accepted. After controlling for the influence of training status, limited structural and functional LV determinants emerged as predictors of ${\dot{V}}_{O_{2}\mathrm{peak}}$. *E*_max_ and *E*_delta_, however, did emerge as significant predictors of ${\dot{V}}_{O_{2}\mathrm{peak}}$ beyond the influence of training status alone. The cumulative effect of all the independent variables in the structural and functional linear mixed effects models at both rest and exercise accounted for approximately 80% of the explained variance in ${\dot{V}}_{O_{2}\mathrm{peak}}$.

### 4.1 Structural predictors of ${\dot{V}}_{O_{2}\mathrm{peak}}$

There was no effect of PWd, VSd, RWT or LV mass index on ${\dot{V}}_{O_{2}\mathrm{peak}}$ in any of the linear mixed effects models. These findings are aligned with previous cross‐sectional, correlational studies in adolescent athletes aged 10–18 years (46% SPs) that demonstrated weak correlations between LV mass index and ${\dot{V}}_{O_{2}\mathrm{peak}}$ (Ujvári et al., 2024). Similar findings were noted with respect to LV volumetric and dimensional attributes. In the present investigation, RWT, a reflection of both LV chamber size and wall thickness, was not a significant predictor of ${\dot{V}}_{O_{2}\mathrm{peak}}$. There is some evidence from the extant literature (Perkins et al., 2022; Forså et al., 2023; Ujvári et al., 2024) with respect to an association between LVEDV and ${\dot{V}}_{O_{2}\mathrm{peak}}$ in pre‐PHV boys, but the strength of these associations (*r* = 0.38–0.67) is variable.

Within the structural linear mixed effects models, the most significant predictor of ${\dot{V}}_{O_{2}\mathrm{peak}}$ was training status. There is previous indirect evidence from longitudinal data obtained by our research team to suggest that the influence of structural and volumetric determinants of ${\dot{V}}_{O_{2}\mathrm{peak}}$ were incorporated within the training stimulus. Unnithan et al. (2024) demonstrated evidence of LV eccentric remodelling in the same group of male SPs used in the present study over a 3‐year period when compared to a group of recreationally active boys tracked over the same time. These authors stated that after controlling for the influence of growth and maturation, there was a training volume threshold that triggered the significant increase in eccentric remodelling seen in years 2 and 3 in the pre‐PHV and circa‐PHV SPs used in the present investigation. Evidence exists within the literature to support this contention (Krustrup et al., 2014; Kinoshita et al., 2015; Weiner et al., 2015; Bjerring et al., 2019). Furthermore, the limited effect of maturity offset as a determinant of ${\dot{V}}_{O_{2}\mathrm{peak}}$ across all the linear mixed effects models allows us to theorise that throughout the 3 years, LV remodelling and adaptation may have occurred as a product of training status rather than growth and maturation to subsequently impact ${\dot{V}}_{O_{2}\mathrm{peak}}$.

In the present investigation, maturity offset (a marker of maturity status) did not emerge as a significant predictor of ${\dot{V}}_{O_{2}\mathrm{peak}}$ in the structural linear mixed effects models in these pre‐ and circa adolescent participants. There is evidence, however, post‐PHV of a greater association between volumetric (LVEDV) and structural (VSd) parameters, respectively, and ${\dot{V}}_{O_{2}\max}$ (Perkins et al., 2022; Forså et al., 2023) compared to trained and untrained pre‐PHV boys. These authors speculated that there appears to be a maturity‐related increased importance of central factors in the oxygen delivery chain with respect to ${\dot{V}}_{O_{2}\max}$ with an increase in biological age. Consequently, the strength and utility of these predictive models using structural independent variables for ${\dot{V}}_{O_{2}\mathrm{peak}}$ were weaker for pre‐PHV boys compared to post‐PHV (Perkins et al., 2022; Forså et al., 2023).

### 4.2 Global functional predictors of ${\dot{V}}_{O_{2}\mathrm{peak}}$ at rest

Training status subsumed effects of any global cardiovascular functional variables [resting heart rate (RHR), LVEF, SVIndex] measured at rest for these pre‐ and circa adolescent participants. Previous evidence (Unnithan et al., 2024) demonstrated training volume induced decrements in LVEF over a 3‐year period that were accompanied by a concomitant increase in LV chamber size in the same SPs as used in the present study. These authors speculated that the training‐induced increase in LV chamber size outweighed the decrease in LVEF over the same period, resulting in a larger SVIndex and a lower RHR. These adaptations created a potential functional reserve for the SP during exercise. The lack of effect of these global markers as predictors of ${\dot{V}}_{O_{2}\mathrm{peak}}$ in the present study was substantiated by findings from trained and untrained pre‐PHV boys, where no global marker of cardiovascular function emerged as independent predictors of ${\dot{V}}_{O_{2}\max}$ (Perkins et al., 2022). Furthermore, weak correlations emerged between SVIndex and ${\dot{V}}_{O_{2}\max}$ in adolescent athletes (Ujvári et al., 2024) aged 10–18 years (46% of whom were SPs).

### 4.3 Tissue‐Doppler predictors of ${\dot{V}}_{O_{2}\mathrm{peak}}$ at submaximal and peak exercise intensities

Training status and body mass emerged as predictors of ${\dot{V}}_{O_{2}\mathrm{peak}}$ across all three submaximal exercise intensities (20, 40 and 60 W). There were no systolic or diastolic TDI predictors at any of the submaximal exercise intensities. Evidence exists, however, to support the contention that there is a significant influence of training on TDI markers during submaximal exercise (Unnithan et al., 2022). In the same group of SPs used in the present study, at the same relative exercise intensity across the three years, superior *E* values were identified in the SPs compared to a group of age matched control participants. In the present study, there appears to be no evidence to suggest that systolic and diastolic markers measured during submaximal exercise have independent predictive power for ${\dot{V}}_{O_{2}\mathrm{peak}}$ beyond the influence of training status. The influence of training status was also noted on *E* in male post‐PHV soccer players, where these athletes presented with superior *E* compared to recreationally active controls at submaximal exercise intensities (Rowland et al., 2009).

Training status, body mass and *E*_max_ emerged as predictors at maximal exercise for ${\dot{V}}_{O_{2}\mathrm{peak}}$. It is possible to speculate that increased *E*_max_ at maximal exercise intensity supports the hypothesis of an increased LVEDV, subsequently an increased SVIndex and increased cardiac output, with consequently sustained peak aerobic capacity despite reduced LV filling time. No inter‐group differences for *E*_max_ were noted between a group of pre‐adolescent SPs and a group of recreationally active controls at peak exercise (Unnithan et al., 2018). This supports the contention that at maximal exercise intensities increased mitral inflow velocities drive ${\dot{V}}_{O_{2}\mathrm{peak}}$, irrespective of training status. The functional reserve (peak − rest) linear mixed effects models identified training status and body mass as significant predictors along with *E*_delta_. This latter finding is aligned with previous work from our research group that identified no significant differences in systolic or diastolic functional reserve markers between pre‐PHV SP and CON groups (Unnithan et al., 2018). It is possible to speculate that at circa‐PHV the training stimulus exerts an effect upon *E*_delta_, and this warrants further investigation.

### 4.4 Strain predictors of ${\dot{V}}_{O_{2}\mathrm{peak}}$ at submaximal intensities

Training status and body mass (across all submaximal intensities) and systolic strain rate at 20 W were identified as significant predictors of ${\dot{V}}_{O_{2}\mathrm{peak}}$. The faster rate of LV shortening during systole at 20 W was related to an increase in ${\dot{V}}_{O_{2}\mathrm{peak}}$. It is possible to speculate that this reflects a greater inotropic effect on contractility at submaximal exercise intensities. Our analyses were limited to global longitudinal strain, but there is evidence to suggest that training status may subsume the influence of circumferential strain on ${\dot{V}}_{O_{2}\mathrm{peak}}$. Previous research has demonstrated in highly trained adolescent SPs (age: 15.4 years), approximately 40% of the variance in ${\dot{V}}_{O_{2}}$ in an incremental exercise test to exhaustion could be explained by circumferential strain (Pieles et al., 2021).

A commonality across several of the linear mixed effects models was that training status was a predictor of ${\dot{V}}_{O_{2}\mathrm{peak}}$ across the three years. It is likely that this variable incorporated a number of the cardiac structural and functional adaptations in response to the training stimulus (Unnithan et al., 2022, 2018, 2024). It is also possible, however, that central determinants of oxygen delivery only emerge as significant factors for ${\dot{V}}_{O_{2}\mathrm{peak}}$ post‐PHV (Perkins et al., 2022). Consequently, there are an array of metabolic adaptations (increased mitochondrial density, oxidative enzymes and H^+^ muscle clearance rate) that occur in response to a training stimulus (Ratel & Blazevich, 2017; Ratel et al., 2008) in a pre‐PHV and circa‐PHV athlete that may also act as predictors of ${\dot{V}}_{O_{2}\mathrm{peak}}$.

### 4.5 Limitations

There were some limitations associated with this study. We did not obtain estimates of fat‐free mass across the 3 years, which would have provided insights on whether lean muscle tissue was a predictor of ${\dot{V}}_{O_{2}\mathrm{peak}}$. Body mass is a surrogate marker of fat free mass within these linear mixed effects models and evidence from the extant literature suggests that fat free mass is a significant independent predictor of ${\dot{V}}_{O_{2}\max}$ in adolescents (Landgraff et al., 2021; Prommer et al., 2018). Test–retest reliability of the key TDI measures was only able to be collected from the SPs in the final year of the study, and we recognise that it would have been better to assess the precision of the findings in both groups across all three years of the study. The study focused on the LV only and the authors acknowledge that acquisition of right ventricular data would have provided greater insights on structural and functional predictors of ${\dot{V}}_{O_{2}\mathrm{peak}}$ across the three years. Furthermore, the impact of training status on LV morphology and function in the SPs and subsequently ${\dot{V}}_{O_{2}\mathrm{peak}}$ could be mediated by the influence of genetic predisposition/self‐selection toward LV adaptation in the SPs.

### 4.6 Conclusion

Training status emerged as the primary significant predictor of ${\dot{V}}_{O_{2}\mathrm{peak}}$ across all longitudinal linear mixed effects models that were developed in this study. The evidence from this novel, 3‐year, longitudinal investigation in a group of trained SPs and recreationally active pre‐PHV males as they transitioned to circa‐PHV is that minimal LV structural and functional adaptations at both rest and exercise influence ${\dot{V}}_{O_{2}\mathrm{peak}}$ beyond the impact of training status alone. The primary influence of LV structural and functional adaptations over 3 years on ${\dot{V}}_{O_{2}\mathrm{peak}}$ may be embedded within the broad training stimulus that highly trained pre‐PHV and circa‐PHV soccer players are exposed to. Indirect evidence exists within our previously published work to support this theory (Unnithan et al., 2024, 2022). The broader implication of these findings is that the influence of cardiac remodelling on ${\dot{V}}_{O_{2}\mathrm{peak}}$ over time is mediated by the stimulus of training; this association occurs independently from the impact of growth and maturation on ${\dot{V}}_{O_{2}\mathrm{peak}}$.

## AUTHOR CONTRIBUTIONS

Viswanath B. Unnithan designed the study, collected data, analysed data and wrote the manuscript; Alexander Beaumont analysed data and edited the manuscript; Thomas Rowland had input into the study design, collected data and edited the manuscript; Keith George had input into the study design and edited the manuscript; Antonio Dello Iacono led the statistical analyses and edited the manuscript; Nicholas Sculthorpe analysed data and edited the manuscript; Rachel N. Lord collected data and edited the manuscript; David L. Oxborough had input into the study design, collected data, analysed data and edited the manuscript. All authors have read and approved the final version of this manuscript and agree to be accountable for all aspects of the work in ensuring that questions related to the accuracy or integrity of any part of the work are appropriately investigated and resolved. All persons designated as authors qualify for authorship, and all those who qualify for authorship are listed.

## CONFLICT OF INTEREST

None declared.

## FUNDING INFORMATION

None.

## Data Availability

Anonymised raw data to view can be accessed at the Open Science Framework using this link: https://osf.io/gt9ks/?view_only=e5680e16f6b245d4af1019b476d5a108
